# Supplementary figures and images for: Insecticidal Activity of Selected Essential Oils against Drosophila suzukii (Diptera: Drosophilidae)
Source: Plants (Basel). 2023 Oct 30;12(21):3727. doi: 10.3390/plants12213727 (PMC10647715; doi:10.3390/plants12213727)

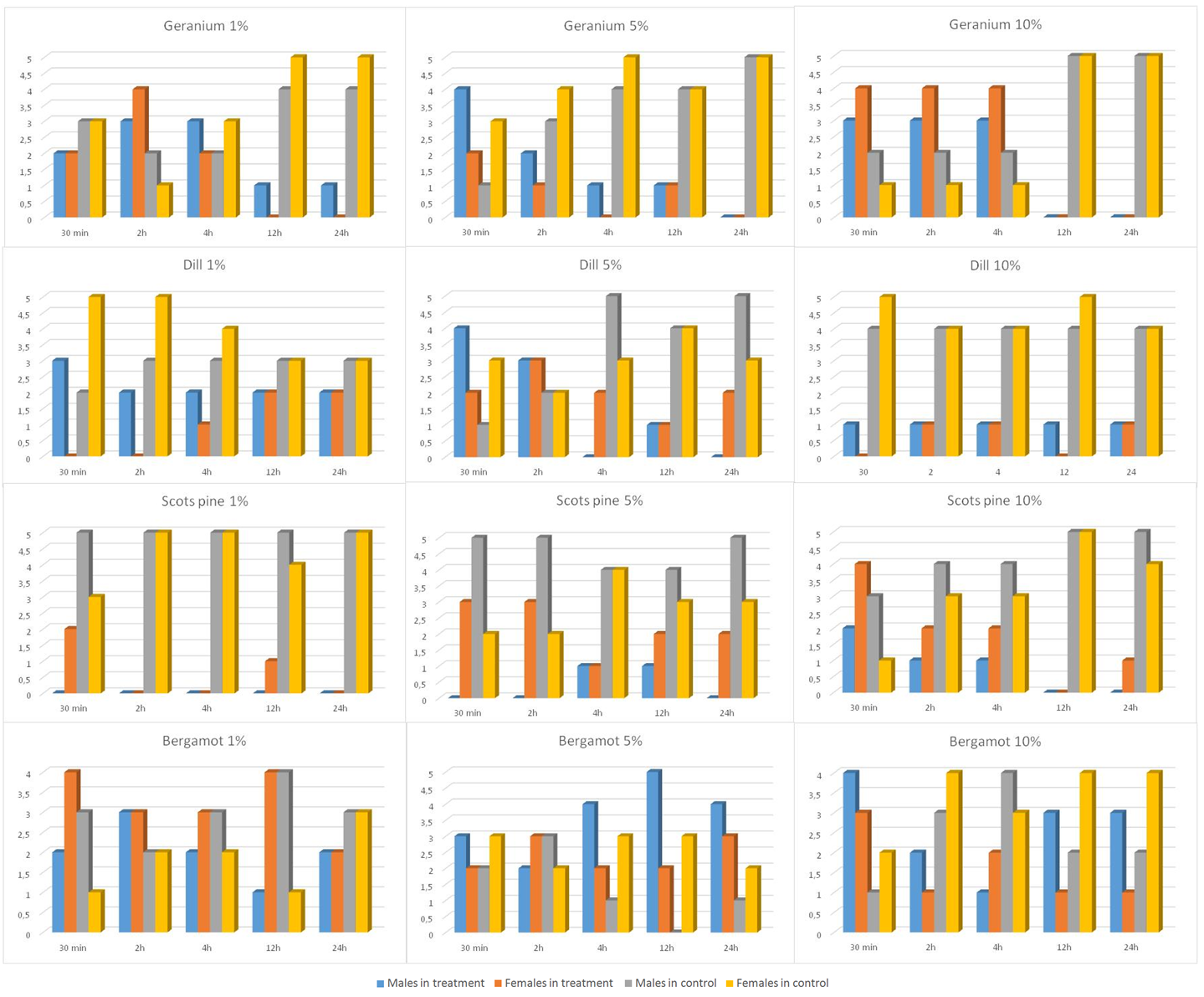

Supplement: Supplementary file 1 [file plants-12-03727-s001.zip › plants-2648495-supplementary.png]
